# Fenestrated endovascular aortic repair of a superior mesenteric artery aneurysm using carbon dioxide angiography and intravascular ultrasound

**DOI:** 10.1016/j.jvscit.2023.101355

**Published:** 2023-10-26

**Authors:** Darren Van Essen, Sarah M. Taylor, Lavraj Lidher, Jeffrey G. Grab, Henry Walton, Jeffery A. Clark

**Affiliations:** a Cumming School of Medicine, University of Calgary, Calgary, AB, Canada; b Faculty of Medicine, University of British Columbia, Vancouver, BC, Canada; c Division of Vascular Surgery, Department of Surgery, University of Calgary, Calgary, AB, Canada; d Department of Radiology, University of Calgary, Calgary, AB, Canada

**Keywords:** Carbon dioxide angiography, Contrast allergy, Fenestrated endovascular aortic repair, Intravascular ultrasound, Superior mesenteric artery aneurysm

## Abstract

Superior mesenteric artery aneurysms are rare; however, current guidelines suggest they all require repair due to the high rupture and mortality rates, and endovascular repair is an effective management strategy. Iodinated contrast traditionally used in endovascular repair can cause significant complications, including severe allergic reactions and contrast-induced nephropathy in patients with chronic renal disease. Therefore, other imaging methods should be used during endovascular procedures to reduce these risks. We describe a unique and innovative approach using carbon dioxide angiography and intravascular ultrasound during fenestrated endovascular repair of an uncommon superior mesenteric artery aneurysm in a patient with severe contrast allergies.

Superior mesenteric artery (SMA) aneurysms (SMAAs) represent 3.5% to 15% of visceral artery aneurysms.^1^, ^2^, ^3^, ^4^ Advances in imaging, including computed tomography angiography, have increased the diagnosis of SMAAs.^1^, ^2^, ^3^, ^4^ Current guidelines suggest all SMAAs require repair due to the high rupture and mortality rates. Open and endovascular surgical procedures are available.^3^^,^^4^ However, fluoroscopy with iodinated contrast during endovascular repair can cause complications, including severe allergic reactions and contrast-induced nephropathy in patients with chronic renal disease. Other imaging methods have been explored to mitigate this risk.^5^, ^6^, ^7^ Carbon dioxide (CO_2_) angiography is a feasible imaging method for these patient populations during endovascular aortic aneurysm repair (EVAR).^6^, ^7^, ^8^, ^9^ However, its use during fenestrated EVAR (FEVAR) remains nearly unexplored.^10^ The role of intravascular ultrasound (IVUS) in these patient populations has also been studied.^11^, ^12^, ^13^ We describe an innovative use of CO_2_ angiography and IVUS during FEVAR of a complex SMAA. Using this unique approach, we eliminated the use of iodinated contrast in a patient with severe contrast allergies. The patient provided written informed consent for the report of his case details and imaging studies.

## Case report

**Fig 1:**
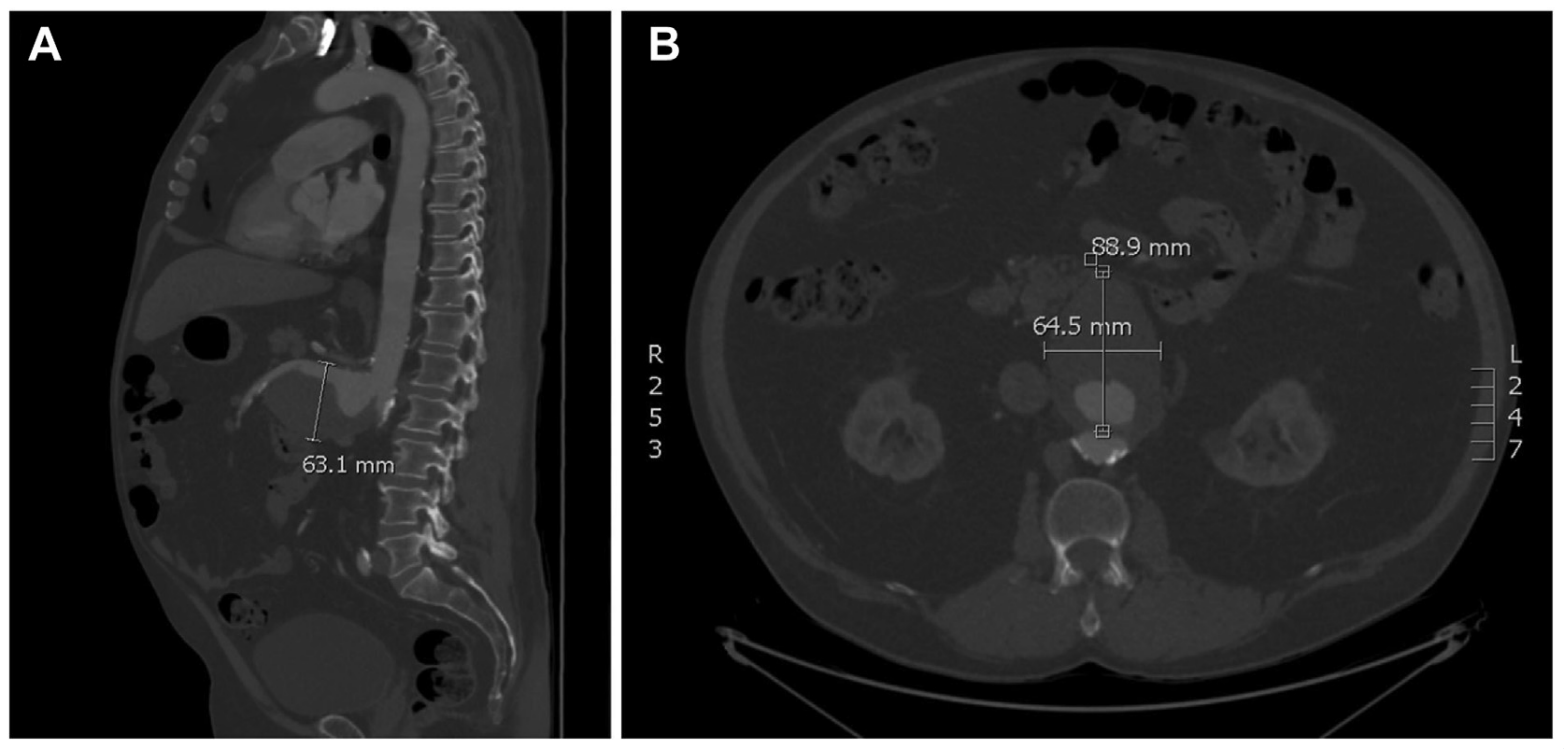
Preoperative sagittal **(A)** and transverse **(B)** computed tomography angiography images of a superior mesenteric artery (SMA) aneurysm (SMAA) measuring 9.6 cm anteroposteriorly, 6.5 cm craniocaudally, and 6.4 cm transversely in the maximal dimensions.

A 67-year-old man underwent endovascular repair of a SMAA with a custom fenestrated aortic graft. The initial computed tomography angiogram revealed a very large saccular SMAA measuring 8.9 cm anteroposteriorly, 6.3 cm craniocaudally, and 6.4 cm transversely (Fig 1). Although the SMA vessel origin was involved, the aorta itself was not aneurysmal along its course.

Endovascular aortic repair was chosen over an open repair given the patient's medical comorbidities, including obesity, hepatitis C, Child Pugh A cirrhosis with previous ascites, hypertension, and hypertriglyceridemia. Because the SMA origin offered no proximal landing zone, a fenestrated graft was selected to provide a proximal seal. CO_2_ angiography and IVUS were chosen as the imaging techniques because of the patient's previously documented anaphylactic reaction to contrast dye, including a pruritic rash, airway swelling, and nausea, despite appropriate premedication.

**Fig 2:**
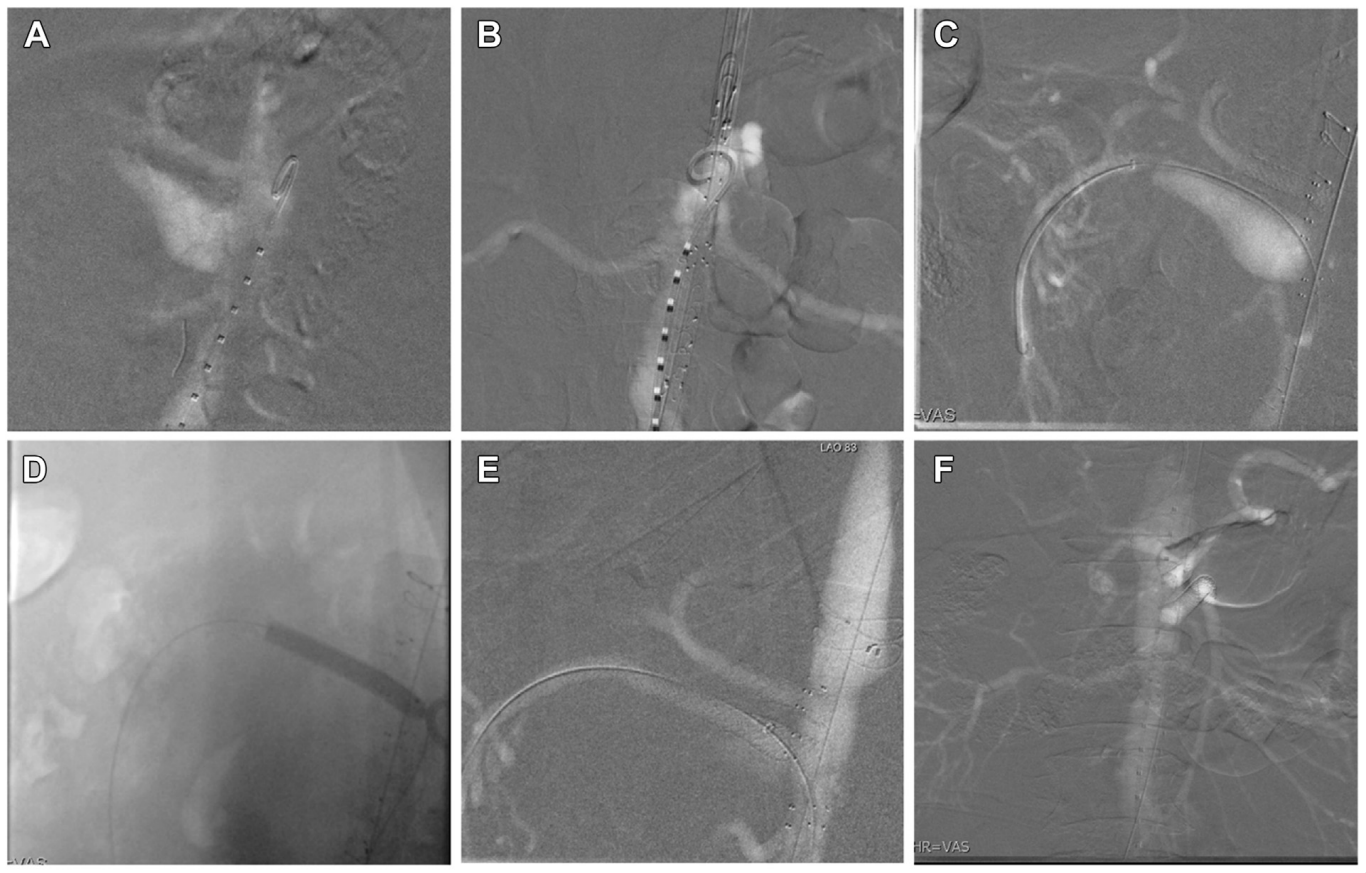
Carbon dioxide (CO_2_) angiography images obtained during fenestrated endovascular repair of the abdominal aorta (FEVAR) for a superior mesenteric artery (SMA) aneurysm (SMAA). **(A)** Sagittal CO_2_ angiography was used to visualize the visceral vessels for main body device placement planning **(B)**. **(C)** A C1 catheter and 8F Destination sheath were used to cross the SMA fenestration and SMAA for deployment of an 8 × 79-mm balloon expandable VBX stent **(D, E)**. **(F)** Completion CO_2_ angiogram demonstrating flow into the visceral abdominal vessels with no obvious endoleak.

**Fig 3:**
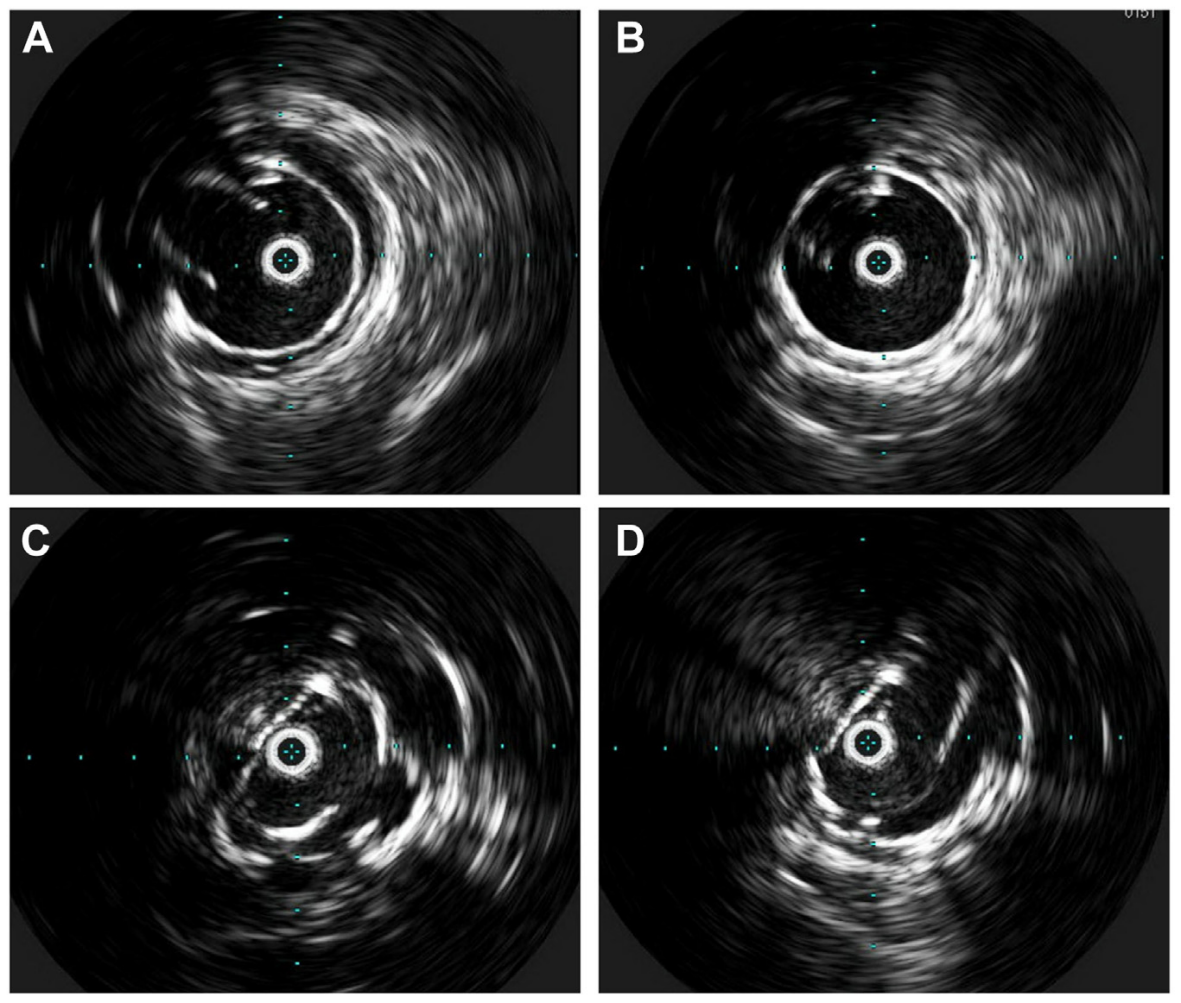
Final intravascular ultrasound (IVUS) imaging confirming visceral artery stent placement. **(A)** Celiac artery. **(B)** Superior mesenteric artery (SMA). **(C)** Left renal artery. **(D)** Right renal artery.

An Anaconda custom fenestrated aortic stent graft (Terumo Aortic; not available in the United States) was placed using CO_2_ angiography using bilateral percutaneous femoral artery access (Fig 2, *A* and *B*). Next, the celiac graft fenestration and artery were cannulated under CO_2_ angiography using a C1 catheter (Cook Medical Inc). An 8 × 38-mm covered balloon-expandable Advanta V12 stent (Atrium Medical, Getinge) was placed and flared proximally into the main body with a 10-mm Mustang balloon (Boston Scientific). CO_2_ angiography confirmed stent placement and flow. Next, the Volcano IVUS imaging system (Philips Healthcare) was used to visualize the remaining visceral branches. The SMA fenestration and SMAA were crossed with the C1 catheter and 8F Destination sheath (Terumo Interventional Systems; Fig 2, *C*). IVUS was used to identify the SMA branch vessels and a landing zone in healthy distal SMA, where an 8 × 79-mm covered balloon expandable VBX stent (Gore Medical) was placed. A second overlapping 8 × 59-mm VBX stent was deployed proximally and flared within the main body (Fig 2, *D*). CO_2_ angiography confirmed stent placement and flow, with sac exclusion (Fig 2, *E*). The renal arteries were similarly cannulated using the C1 catheter and an 8F Destination sheath and reconstructed using 6 × 38-mm Advanta V12 stents bilaterally flared in the fenestrated graft. Completion CO_2_ angiography demonstrated good flow into the celiac artery, SMA, and renal arteries with no endoleak (Fig 2, *F*). Finally, IVUS confirmed appropriate placement and flaring of all the stents (Fig 3). Overall, the fluoroscopic time was 39.5 minutes, and 0 mL of contrast dye was used.

Postoperative day 2 magnetic resonance angiography demonstrated celiac artery, SMA, and bilateral renal artery stent graft patency with no significant endoleaks. Contrast-enhanced ultrasound at 6 months postoperatively showed no evidence of an endoleak with effective aneurysm sac exclusion and normal endograft filling.

## Discussion

SMAAs represent 3.5% to 15% of visceral artery aneurysms. Most arise in the proximal 5 cm of the artery, and ∼63% to 64% occur in men.^1^, ^2^, ^3^ In the present case, the saccular aneurysm, >6 cm in size, involved the SMA origin (Fig 1). Although older literature suggests most SMAAs were of mycotic etiology, recent evidence suggests most are degenerative.^1^^,^^2^ Other risk factors for SMAA development include aging, atherosclerosis, connective tissue disease, inflammatory conditions, trauma, and fibromuscular dysplasia.^1^^,^^2^^,^^14^^,^^15^ Our patient had no identified history of pancreatitis and a remote history of sepsis secondary to severe pneumonia. A preoperative white blood cell scan demonstrated no abnormal activity in the aneurysm, making a degenerative etiology more likely than a mycotic etiology. Severe complications of SMAAs include rupture, mortality, thrombosis, and distal embolization causing intestinal ischemia.^1^^,^^2^^,^^14^^,^^15^ Recent data suggest that splanchnic artery aneurysms ≤2.0 to 2.5 cm in size are safe to observe^2^^,^^14^^,^^15^; however, the current Society for Vascular Surgery guidelines recommend operative repair due to the high rupture rates.^3^ Although asymptomatic, the large aneurysmal size and saccular features were indications for repair in our case.

The options for open surgical SMAA repair include ligation, aneurysmorrhaphy, interposition bypass, and aortic–mesenteric bypass grafting.^2^^,^^4^ Endovascular options include coil embolization and stenting.^2^^,^^4^^,^^16^ Despite the benefits of endovascular management, the aneurysm anatomy, pathogenesis, patient comorbidities, and surgical history dictate the management approach.^4^^,^^16^ Our patient’s medical comorbidities were a significant factor in choosing an endovascular approach in this case. However, contraindications to iodinated contrast medium for intraoperative imaging (ie, severe allergic reactions or contrast-induced nephropathy in patients with chronic renal disease) must be considered.^5^, ^6^, ^7^ Our patient had a prior documentation of an anaphylactic reaction to contrast despite appropriate premedication.

Although infrequent, imaging methods without iodinated contrast, including CO_2_ angiography, have been studied during endovascular aortic procedures. Specifically, CO_2_ angiography during EVAR was associated with decreased renal toxicity, while maintaining technical success, morbidity, and mortality rates similar to those for procedures performed with iodinated contrast.^8^^,^^9^^,^^11^ The use of IVUS during EVAR can also reduce the need for iodinated contrast. Furthermore, IVUS is valuable during the intraoperative stent landing zone assessment and graft placement.^11^, ^12^, ^13^ The usage of CO_2_ angiography and IVUS during FEVAR, however, remains nearly unexplored.

Our innovative technique demonstrates that CO_2_ angiography and IVUS can function as complementary tools during FEVAR. Using these tools, a uniquely large and complex SMAA was successfully repaired with FEVAR without the use of any iodinated contrast medium. Only one recent study has compared the combined use of CO_2_ angiography and fluoroscopy vs fluoroscopy alone during FEVAR.^10^ The addition of CO_2_ angiography lowered the rates of postoperative renal insults, with better preserved renal function at 30 days, decreased contrast medium usage, and shortened hospital stays.^10^ Newer research suggests that the benefits of CO_2_ angiography on renal function are not limited to the postoperative period, with a greater degree of preservation of renal function at 1 year after EVAR.^17^ Another study demonstrated that CO_2_ angiography is especially helpful for identifying the patency and position of target vessels when misaligned with stent fenestrations.^18^ The evidence from these studies, and the success in this innovative technique, demonstrate that performing this procedure without the use of iodinated contrast expands the current treatment options for patients with contraindications to contrast.

## Conclusions

This case demonstrates the successful use of CO_2_ angiography and IVUS during FEVAR of a uniquely large and complex SMAA. With this novel approach, we eliminated the need for iodinated contrast in a patient with severe allergies. Our technique not only demonstrates that CO_2_ angiography and IVUS are useful options during FEVAR but also expands the options for patients with contraindications to contrast medium.

## Disclosures

None.
